# Luteal blood flow and luteal function

**DOI:** 10.1186/1757-2215-2-1

**Published:** 2009-01-14

**Authors:** Akihisa Takasaki, Hiroshi Tamura, Ken Taniguchi, Hiromi Asada, Toshiaki Taketani, Aki Matsuoka, Yoshiaki Yamagata, Katsunori Shimamura, Hitoshi Morioka, Norihiro Sugino

**Affiliations:** 1 Department of Obstetrics and Gynecology, Yamaguchi University Graduate School of Medicine, Minamikogushi 1-1-1, Ube, 755-8505 Japan; 2 Department of Obstetrics and Gynecology, Saiseikai Shimonoseki General Hospital, Kifunecho 3-1-37, Shimonoseki, 751-0823, Japan

## Abstract

**Background:**

Blood flow in the corpus luteum (CL) is associated with luteal function. The present study was undertaken to investigate whether luteal function can be improved by increasing CL blood flow in women with luteal phase defect (LFD).

**Methods:**

Blood flow impedance in the CL was measured by transvaginal color-pulsed-Doppler-ultrasonography and was expressed as a resistance index (RI). The patients with both LFD [serum progesterone (P) concentrations < 10 ng/ml during mid-luteal phase] and high CL-RI (≥ 0.51) were given vitamin-E (600 mg/day, n = 18), L-arginine (6 g/day, n = 14) as a potential nitric oxide donor, melatonin (3 mg/day, n = 13) as an antioxidant, or HCG (2,000 IU/day, n = 10) during the subsequent menstrual cycle.

**Results:**

In the control group (n = 11), who received no medication to increase CL blood flow, only one patient (9%) improved in CL-RI and 2 patients (18%) improved in serum P. Vitamin-E improved CL-RI in 15 patients (83%) and improved serum P in 12 patients (67%). L-arginine improved CL-RI in all the patients (100%) and improved serum P in 10 patients (71%). HCG improved CL-RI in all the patients (100%) and improved serum P in 9 patients (90%). Melatonin had no significant effect.

**Conclusion:**

Vitamin-E or L-arginine treatment improved luteal function by decreasing CL blood flow impedance. CL blood flow is a critical factor for luteal function.

## Background

During corpus luteum formation, active angiogenesis occurs after the ovulatory LH surge, and the corpus luteum becomes one of the most highly vascularized organs in the body [1-7]. Blood flow in the corpus luteum is important for the development of the corpus luteum and maintenance of luteal function [7-12]. Adequate blood flow in the corpus luteum is necessary to provide luteal cells with the large amounts of cholesterol that are needed for progesterone synthesis and to deliver progesterone to the circulation. Color Doppler ultrasonography is a useful and noninvasive technique for evaluating ovarian vascular function, allowing visual observation of the blood flow within the corpus luteum [13-16]. Blood flow in the corpus luteum measured by color Doppler ultrasonography is well associated with luteal function [13-21].

We recently reported changes in blood flow in the human corpus luteum throughout the luteal phase and a close relationship between luteal blood flow and luteal function [16]. Interestingly, luteal blood flow was significantly correlated with serum progesterone concentrations during the mid-luteal phase, and luteal blood flow was significantly lower in women with luteal phase defect than in women with normal luteal function, suggesting that low blood flow of the corpus luteum is associated with luteal phase defect. We, therefore, decided to study whether luteal phase defect can be improved by increasing luteal blood flow.

For this purpose, we focused on vitamin E and a potential nitric oxide (NO) donor, L-arginine, to increase luteal blood flow. Vitamin E has been shown to improve capillary blood flow in a variety of organs not only by inhibiting the breakdown of lipids in red blood cell membranes [22,23] but also by protecting the endothelium from oxidative stress [24,25]. NO release by vascular endothelial cells via endothelial NO synthase (eNOS) leads to the relaxation of vascular smooth muscle, mainly by activating cyclic guanosine monophosphate (cGMP) [26]. L-arginine, a substrate of NO, increases hepatic and limb blood flow [27,28]. In the present study, in order to examine the role of luteal blood flow in the regulation of luteal function, we investigated whether luteal function can be improved by increasing luteal blood flow in patients with luteal phase defect.

It has also been reported that decreased blood flow causes oxidative stress in a variety of organs [29-31]. Oxidative stress is well known to inhibit luteal function [30,31]. In fact, the decrease in ovarian blood flow inhibits luteal function through oxidative stress in rats [29]. Therefore, we further examined the possibility that the decrease in blood flow of the corpus luteum inhibits luteal function through oxidative stress in patients with luteal phase defect.

## Methods

The project was reviewed and approved by Institutional Review Board of Yamaguchi University Graduate School of Medicine. Informed consent was obtained from all the patients in this study.

### Patients

A total of 66 women who had both luteal phase defect and high blood flow impedance of the corpus luteum [corpus luteum-resistance index (CL-RI) ≥ 0.51] were recruited into this study. When serum progesterone concentrations were < 10 ng/ml during the mid-luteal phase, the patient was diagnosed as having a luteal phase defect in this study. CL-RI was measured during the mid-luteal phase, and the cutoff value was determined as described below. The mean age was 32.4 ± 4.3 years (mean ± SD), with a range of 24–41 years. The patients were non-smokers and free from major medical illness including hypertension; they were excluded if they had myoma, adenomyosis, congenital uterine anomaly, or ovarian tumors or if they used estrogens, progesterone, androgens, or had chronic use of any medication, including nonsteroidal anti-inflammatory agents or anticonvulsants.

### Ultrasonography

Blood flow in the corpus luteum was measured as reported previously [16] using a computerized ultrasonography with an integrated pulsed Doppler vaginal scanner [Aloka ProSound SSD-3500SV and Aloka UST-984-5 (5.0 MHz) vaginal transducer, Aloka Co. Ltd, Tokyo, Japan]. The high pass filter was set at 100 Hz, and the pulse repetition frequency was 2–12 kHz, for all Doppler spectral analyses. After the endovaginal probe was gently inserted into the vagina, adnexal regions were thoroughly scanned. The ovary was identified, and color signals were used to detect the area with the highest blood flow within the corpus luteum. Blood flow was identified in the peripheral area of the corpus luteum [16]. The pulsed Doppler gate was then placed on that area to obtain flow velocity waveforms. An acceptable angle was less than 60°, and the signal was updated until at least four consecutive flow velocity waveforms of good quality were obtained. Blood flow impedance was estimated by calculating the resistance index (RI), which is defined as the difference between maximal systolic blood flow (S) and minimal diastolic flow (D) divided by the peak systolic flow (S-D/S). Blood flow impedances were examined in the corpus luteum during the mid-luteal phase (6–8 days after ovulation). The day of ovulation was determined by urinary LH, transvaginal ultrasonography and basal body temperature records. Since the interobserver coefficient of variation for Doppler flow measurements in the present study was less than 10%, which is consistent with the reports by Ziegler *et al*. [32] and Miwa *et al*. [33], the Doppler flow measurements were judged to be reproducible.

**Figure 1:**
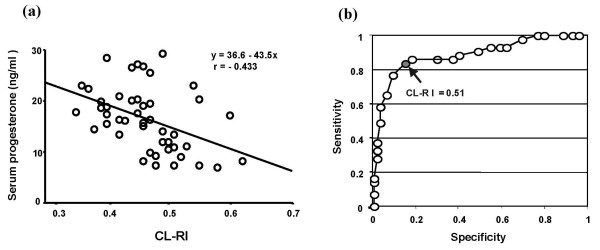
**Correlation between blood flow impedance of the corpus luteum and serum progesterone concentrations**. (a): Correlation between corpus luteum-resistance index (CL-RI) and serum progesterone concentrations (n = 46). (b): Receiver operating characteristic (ROC) curve analysis. CL-RI and serum progesterone concentrations were measured during the mid-luteal phase (6–8 days after ovulation). Serum progesterone concentrations were significantly and negatively correlated with CL-RI (p < 0.01, single regression analysis). ROC curve analysis was performed to determine the cutoff value of the CL-RI providing the best values of sensitivity and specificity for determination of normal luteal function and luteal phase defect. The cutoff value of 0.51 provided the best combination with 84.3% sensitivity and 85.6% specificity to discriminate between normal luteal function and luteal phase defect.

There was a significant negative correlation between CL-RI and serum progesterone concentrations during the mid-luteal phase from the data obtained from 36 women with normal luteal function and 10 women with luteal phase defect (Fig. 1a). Receiver operating characteristic curve (ROC) analysis was performed to determine the cutoff value of the CL-RI providing the best value of the sensitivity and the specificity for determination of normal luteal function and luteal phase defect. A cutoff value of 0.51 provided the best combination with 84.3% sensitivity and 85.6% specificity to discriminate between normal luteal function and luteal phase defect (Fig. 1b).

### Clinical studies

In order to investigate whether vitamin E or L-arginine treatment has a potential to increase luteal blood flow and to improve luteal function in patients with luteal phase defect, the patients who showed both luteal phase defect and high CL-RI (≥ 0.51) during the mid-luteal phase (6–8 days after ovulation) were given vitamin E (600 mg/day, 3 times per day orally; Eisai Co., Ltd., Tokyo, Japan; n = 18), or L-arginine (6 g/day, 4 times per day orally; Now Foods, IL, USA; n = 14) during the luteal phase of the subsequent menstrual cycle.

Decreased ovarian blood flow is reported to inhibit luteal function via oxidative stress [29]. Therefore, to examine a possibility that the decrease in luteal blood flow inhibits luteal function through oxidative stress in women with luteal phase defect, melatonin (3 mg at 22:00 hr orally; KAL, Park City, UT, USA; n = 13) was given as an antioxidant during the luteal phase of the subsequent menstrual cycle. We confirmed that administration of 3 mg of melatonin works as an antioxidant and suppresses oxidative stress in the human ovulatory follicle [34].

Another 10 patients received luteal support with HCG injection (2,000 IU/day, on days 3 and 5 after ovulation; Gonatropin; Asuka Co., Ltd., Tokyo, Japan).

As controls, 11 patients with both luteal phase defect and high CL-RI (≥ 0.51) during the mid-luteal phase received no medication during the subsequent menstrual cycle.

To evaluate the effect of those treatments, serum progesterone concentrations and CL-RI were measured during the mid-luteal phase (6–8 days after ovulation). Ultrasonogrphy was performed before blood sampling for serum progesterone measurement.

### Progesterone assay

Venous blood was taken for the determination of serum progesterone concentrations on the day of the Doppler examination during the mid-luteal phase. Progesterone concentrations were measured by enzyme immunoassay (ST AIA-PACK PROG, Tosoh Co., Ltd., Japan) as reported previously [16]. The minimal detectable concentration is estimated to be 0.1 ng/ml. Intra-assay and inter-assay coefficients of variation were 9.9% and 11.3%, respectively.

### Statistical analyses

Single regression analysis, Wilcoxon signed-ranks test, and chi-squared test with Bonferroni correction were carried out using the computer program SPSS for windows 13.0. A value of P < 0.05 was considered significant.

## Results

### Vitamin E treatment

Eighteen patients who had both luteal phase defect and high CL-RI (≥ 0.51) during the mid-luteal phase were given vitamin E during the luteal phase of the subsequent menstrual cycle. Fifteen patients out of 18 (83%) showed improved CL-RI of less than 0.51, and 12 patients (67%) developed serum progesterone concentrations of more than 10 ng/ml (Table 1). In the control group, only one patient out of 11 (9%) showed normal CL-RI and 2 patients (18%) showed normal serum progesterone concentrations (Table 1), suggesting that vitamin E significantly improved CL-RI and serum progesterone concentrations compared with the control (Table 1). Of the 12 patients whose serum progesterone concentrations improved, 11 patients showed improved CL-RI of less than 0.51. Vitamin E significantly decreased CL-RI and increased serum progesterone concentrations between the treatment cycle and the previous cycle (Table 1).

**Table 1:** Effects of vitamin E, L-arginine, melatonin, or HCG on corpus luteum resistance index and serum progesterone concentrations in patients with luteal phase defect.

			**CL-RI**		**Serum P (ng/ml)**		
	n	previous cycle	treatment cycle	No. of < 0.51	previous cycle	Treatment cycle	No. of ≥ 10 ng/ml

**Control**	11	0.544 (0.515–0.643)	0.552 (0.483–0.633)	1 (9%)	7.2 (4.5–9.7)	8.2 (6.1–16.7)	2 (18%)

**Vitamin E**	18	0.550 (0.514–0.632)	0.448^a ^(0.376–0.681)	15 (83%)^c^	8.0 (5.8–9.2)	11.6^a ^(6.4–21.6)	12 (67%)^d^

**L-arginine**	14	0.538 (0.513–0.676)	0.419^a ^(0.348–0.483)	14 (100%)^c^	7.6 (2.4–9.4)	12.8^a ^(6.5–22.8)	10 (71%)^d^

**Melatonin**	13	0.538 (0.515–0.676)	0.530 (0.431–0.691)	4 (31%)	7.7 (2.4–8.9)	9.5^b ^(2.9–29.1)	5 (38%)

**HCG**	10	0.545 (0.518–0.931)	0.447^a ^(0.406–0.506)	10 (100%)^c^	8.1 (5.9–9.2)	14.7^a ^(8.8–18.4)	9 (90%)^c^

Sixty-six patients with both luteal phase defect and high corpus luteum-resistance index (CL-RI ≥ 0.51) were recruited in this study. Vitamin E (600 mg/day, n = 18), L-arginine (6 g/day, n = 14), or melatonin (3 mg/day, n = 13) was given after ovulation throughout the luteal phase. Controls received no medication (n = 11). Ten patients received luteal support with HCG (2,000 IU/day, on days 3 and 5 after ovulation). Data were compared between the treatment cycle and the previous cycle in each treatment, and between the control group and each treatment group. One patient out of 11 (9%) spontaneously improved in CL-RI and 2 patients (18%) did in serum progesterone (P) in the control group. By vitamin E treatment, 15 patients out of 18 (83%) showed improved CL-RI, 12 patients (67%) developed a serum P of more than 10 ng/ml. L-arginine treatment improved CL-RI in all the patients (100%) and serum P in 10 patients out of 14 (71%). Melatonin treatment had no significant effect on CL-RI. HCG treatment improved CL-RI in all the patients (100%) and serum P in 9 patients out of 10 (90%). Values show median with ranges. a; p < 0.01 and b; p < 0.05 v.s. previous cycle (Wilcoxon test). c; p < 0.01 and d; p < 0.05 v.s. control (x^2^-test with Bonferroni correction).

### L-arginine treatment

L-arginine treatment improved CL-RI in all the patients (100%), and 10 patients out of 14 (71%) developed serum progesterone concentrations of more than 10 ng/ml (Table 1). Compared with the control, L-arginine significantly improved CL-RI and serum progesterone concentrations (Table 1). L-arginine also significantly decreased CL-RI and increased serum progesterone concentrations between the treatment cycle and the previous cycle (Table 1).

### Melatonin treatment

Melatonin treatment improved CL-RI in 4 patients out of 13 (31%) and improved serum progesterone concentrations in 5 patients (38%) (Table 1). These effects were not significant compared with the control (Table 1). Melatonin treatment caused a significant increase in serum progesterone concentrations in the treatment cycle compared with the previous cycle, but the serum progesterone levels were less than 10 ng/ml (Table 1).

### HCG treatment

HCG treatment improved CL-RI in all the patients (100%), and 9 patients out of 10 (90%) developed serum progesterone concentrations of more than 10 ng/ml (Table 1). Compared with the control, HCG significantly improved CL-RI and serum progesterone concentrations (Table 1). HCG also significantly decreased CL-RI and increased serum progesterone concentrations between the treatment cycle and the previous cycle (Table 1).

## Discussion

Luteal phase defect has been implicated as a cause of infertility and spontaneous miscarriage. Previous reports including our recent report suggest that luteal phase defect is associated with high blood flow impedance of the corpus luteum, because luteal blood flow impedance in women with luteal phase defect during the mid-luteal phase was significantly higher than it was in women with normal luteal function [13,14,16,19,20], and CL-RI was negatively correlated with serum progesterone concentrations during the mid-luteal phase. The present study showed that treatments with vitamin E or L-arginine significantly improved CL-RI and luteal function in patients with luteal phase defect and high CL-RI. Most of the patients whose CL-RI was improved by vitamin E or L-arginine showed improvement of luteal function. Furthermore, in our unpublished data, administration of progesterone as a luteal support for the patients with both luteal phase defect and high CL-RI did not improve CL-RI during the mid-luteal phase, suggesting that progesterone does not influence luteal blood flow impedance. It is, therefore, likely that vitamin E or L-arginine improves luteal function by decreasing luteal blood flow impedance. The present result that decreasing luteal blood flow impedance improved luteal function strongly suggests that high blood flow impedance of the corpus luteum is involved in the pathophysiology of impaired luteal function in patients with luteal phase defect. In other words, luteal blood flow is a critical factor for luteal function.

Although there are no well-established methods for increasing luteal blood flow, the present results appear to be consistent with previous reports by ourselves and others that vitamin E, L-arginine, or sildenafil citrate (Viagra) improved endometrial growth in patients with a thin endometrium by increasing uterine artery blood flow [33,35-37].

Decreased ovarian blood flow is reported to inhibit luteal function via oxidative stress [29]. Oxidative stress is well known to inhibit luteal function [30,31]. It is of interest to note that vitamin E acts as an antioxidant. The present result revealed that melatonin used as an antioxidant did not improve luteal blood flow impedance or luteal function, suggesting that vitamin E works via decreasing luteal blood flow impedance rather than by acting as an antioxidant.

Interestingly, HCG improved luteal blood flow impedance as well as L-arginine. Although it is unclear how HCG increases luteal blood flow, it may work through vasoactive substances because there is some evidence that luteal phase defect is caused by the altered regulation of luteal blood flow during the mid-luteal phase [16]. Vasoactive substances such as NO, endothelin, or angiotensin have been reported to be involved in luteal function [11,38-41]. HCG increases eNOS expression in the ovary of the rat and sheep [42,43], and increases rat ovarian blood flow via locally produced NO [44]. Further studies are needed to elucidate the relationship between luteal blood flow and vasoactive substances in the corpus luteum.

## Conclusion

The present study is, to our knowledge, the first report to show a close relationship between luteal blood flow impedance, luteal function, and treatments that improve luteal blood flow. Treatments that improve luteal blood flow seem to improve luteal function in patients with both luteal phase defect and high luteal blood flow impedance. In other words, luteal blood flow is a critical factor for luteal function. However, the present study is a pilot study with a small number of subjects. A prospective randomized controlled trial with larger samples is needed to demonstrate the efficacy of these treatments for luteal phase defect.

## Competing interests

The authors declare that they have no competing interests.

## Authors' contributions

AT conceived of the study, participated in its design, collected the data, and prepared the original manuscript. HT, KT, HA, TT, AM, YY, KS and HM collected the data. NS conceived of the study, participated in its design, drafted the final manuscript, and directed the research. All authors approved the final manuscript.

## References

[B1] Ferrara N, Chen H, Davis-Smyth T, Geber HP, Nguyen TN, Peers D, Chisholm V, Hillan K, Schwall R (1998). Vascular endothelial growth factor is essential for corpus luteum angiogenesis. Nat Med.

[B2] Suzuki T, Sasano H, Takaya R, Fukaya T, Yajima A, Nagura H (1998). Cyclic changes of vasculature and vascular phenotypes in normal human ovaries. Hum Reprod.

[B3] Hazzard TM, Stouffer RL (2000). Angiogenesis in ovarian follicular and luteal development. Baillieres Best Pract Res Clin Obstet Gynaecol.

[B4] Fraser HM, Dickson SE, Lunn SF, Wulff C, Morris KD, Carroll VA, Bicknell R (2000). Suppression of luteal angiogenesis in the primate after neutralization of vascular endothelial growth factor. Endocrinology.

[B5] Sugino N, Kashida S, Takiguchi S, Karube A, Kato H (2000). Expression of vascular endothelial growth factor and its receptors in the human corpus luteum during the menstrual cycle and in early pregnancy. J Clin Endocrinol Metab.

[B6] Sugino N, Suzuki T, Sakata A, Miwa I, Asada H, Taketani T, Yamagata Y, Tamura H (2005). Angiogenesis in the human corpus luteum: changes in expression of angiopoietins in the corpus luteum throughout the menstrual cycle and in early pregnancy. J Clin Endocrinol Metab.

[B7] Sugino N, Matsuoka A, Taniguchi K, Tamura H (2008). Angiogenesis in the human corpus luteum. Reprod Med Biol.

[B8] Niswender GD, Reimers TJ, Diekman MA, Nett TM (1976). Blood flow: a mediator of ovarian function. Biol Reprod.

[B9] Wiltbank MC, Dysko RC, Gallagher KP, Keyes PL (1988). Relationship between blood flow and steroidogenesis in the rabbit corpus luteum. J Reprod Fertil.

[B10] Kashida S, Sugino N, Takiguchi S, Karube A, Takayama H, Yamagata Y, Nakamura Y, Kato H (2001). Regulation and role of vascular endothelial growth factor in the corpus luteum during mid-pregnancy in rats. Biol Reprod.

[B11] Miyamoto A, Shirasuna K, Wijayagunawardane MP, Watanabe S, Hayashi M, Yamamoto D, Matsui M, Acosta TJ (2005). Blood flow: a key regulatory component of corpus luteum function in the cow. Domest Anim Endocrinol.

[B12] Matsuoka-Sakata A, Tamura H, Asada H, Miwa I, Taketani T, Yamagata Y, Sugino N (2006). Changes in vascular leakage and expression of angiopoietins in the corpus luteum during pregnancy in rats. Reproduction.

[B13] Kupesic S, Kurjak A (1997). The assessment of normal and abnormal luteal function by transvaginal color Doppler sonography. Eur J Obstet Gynecol Reprod Biol.

[B14] Miyazaki T, Tanaka M, Miyakoshi K, Minegishi K, Kasai K, Yoshimura Y (1998). Power and colour Doppler ultrasonography for the evaluation of the vasculature of the human corpus luteum. Hum Reprod.

[B15] Ottander U, Solensten NG, Bergh A, Olofsson JI (2004). Intraovarian blood flow measured with color Doppler ultrasonography inversely correlates with vascular density in the human corpus luteum of the menstrual cycle. Fertil Steril.

[B16] Tamura H, Takasaki A, Taniguchi K, Matsuoka A, Shimamura K, Sugino N (2008). Changes in blood flow impedance of the human corpus luteum throughout the luteal phase and during early pregnancy. Fertil Steril.

[B17] Alcazar JL, Laparte C, Lopez-Garcia G (1996). Corpus luteum blood flow in abnormal early pregnancy. J Ultrasound Med.

[B18] Bourne TH, Hagstrom H, Hahlin M, Josefsson B, Granberg S, Hellberg P, Hamberger L, Collins WP (1996). Ultrasound studies of vascular and morphological changes in the human corpus luteum during the menstrual cycle. Fertil Steril.

[B19] Glock JL, Brumsted JR (1996). Color flow pulsed Doppler ultrasound in diagnosing luteal phase defect. Fertil Steril.

[B20] Kalogirou D, Antoniou G, Botsis D, Kontoravdis A, Vitoratos N, Giannikos L (1997). Transvaginal Doppler ultrasound with color flow imaging in the diagnosis of luteal phase defect (LPD). Clin Exp Obstet Gynecol.

[B21] Merce LT, Bau S, Bajo JM (2001). Doppler study of arterial and venous intraovarian blood flow in stimulated cycles. Ultrasound Obstet Gynecol.

[B22] Chung TW, Chen TZ, Yu JJ, Lin SY, Chen SC (1995). Effects of α-tocopherol nicotinate on hemorheology and retinal capillary blood flow in female NIDDM with retinopathy. Clin Hemorheol.

[B23] Chung TW, Yu JJ, Liu DZ (1998). Reducing lipid peroxidation stress of erythrocyte membrane by α-tocopherol nicotinate plays an important role in improving blood rheological properties in type 2 diabetic patients with retinopathy. Diabetic Med.

[B24] Shimpuku H, Tachi Y, Shinohara M, Ohura K (2000). Effect of vitamin E on the degradation of hydrogen peroxide in cultured human umbilical vein endothelial cells. Life Sci.

[B25] Huang J, de Paulis T, May JM (2004). Antioxidant effects of dihydrocaffeic acid in human EA.hy926 endothelial cells. J Nutr Biochem.

[B26] Moncada S, Higgs EA (1993). The L-arginine-nitric oxide pathway. N Engl J Med.

[B27] Bode-Boger SM, Boger RH, Alfke H, Heinzel D, Tsikas D, Creutzig A (1996). L-arginine induces nitric oxide-dependent vasodilatation in patients with critical limb ischemia: a randomized, controlled study. Circulation.

[B28] Vertiz-Hernandez A, Castaneda-Hernandez G, Martinez-Cruz A, Cruz-Antonio L, Grijalva I, Guizar-Sahagun G (2007). L-arginine reverses alterations in drug disposition induced by spinal cord injury by increasing hepatic blood flow. J Neurotrauma.

[B29] Sugino N, Nakamura Y, Okuno N, Ishimatsu M, Teyama T, Kato H (1993). Effects of ovarian ischemia-reperfusion on luteal function in pregnant rats. Biol Reprod.

[B30] Sugino N (2005). Reactive oxygen species in ovarian physiology. Reprod Med Biol.

[B31] Sugino N (2006). Roles of reactive oxygen species in the corpus luteum. Animal Science Journal.

[B32] Ziegler WF, Bernstein I, Badger G, Leavitt T, Cerrero ML (1999). Regional hemodynamic adaptation during the menstrual cycle. Obstet Gynecol.

[B33] Miwa I, Tamura H, Takasaki A, Yamagata Y, Shimamura K, Sugino N (2009). Pathophysiological features of thin endometrium. Fertil Steril.

[B34] Tamura H, Takasaki A, Miwa I, Taniguchi K, Maekawa R, Asada H, Taketani T, Matsuoka A, Yamagata Y, Shimamura K, Morioka H, Ishikawa H, Reiter RJ, Sugino N (2008). Oxidative stress impairs oocyte quality and melatonin protects oocytes from free radical damage and improves fertilization rate. J Pineal Res.

[B35] Lédée-Bataille N, Olivennes F, Lefaix JL, Chaouat G, Frydman R, Delanian S (2002). Combined treatment by pentoxifylline and tocopherol for recipient women with a thin endometrium enrolled in an oocyte donation programme. Hum Reprod.

[B36] Sher G, Fisch JD (2000). Vaginal sildenafil (Viagra): a preliminary report of a novel method to improve uterine artery blood flow and endometrial development in patients undergoing IVF. Hum Reprod.

[B37] Sher G, Fisch JD (2002). Effect of vaginal sildenafil on the outcome of in vitro fertilization (IVF) after multiple IVF failures attributed to poor endometrial development. Fertil Steril.

[B38] Apa R, Miceli F, de Feo D, Pierro E, Ayaia G, Mancuso S, Napolitano M, Lanzone A (1998). Endothelin-1: expression and role in human corpus luteum. Am J Reprod Immunol.

[B39] Tognetti T, Estevez A, Luchetti CG, Sander V, Franchi AM, Motta AB (2003). Relationship between endothelin-1 and nitric oxide system in the corpus luteum regression. Prostaglandins Leukot Essent Fatty Acids.

[B40] Klipper E, Gilboa T, Levy N, Kisliouk T, Spanel-Borowski K, Meidan R (2004). Characterization of endothelin-1 and nitric oxide generating systems in corpus luteum-derived endothelial cells. Reproduction.

[B41] Rosiansky-Sultan M, Klipper E, Spanel-Borowski K, Meidan R (2006). Inverse relationship between nitric oxide synthases and endothelin-1 synthesis in bovine corpus luteum: interactions at the level of luteal endothelial cell. Endocrinology.

[B42] Nakamura Y, Kashida S, Nakata M, Takiguchi S, Yamagata Y, Takayama H, Sugino N, Kato H (1999). Changes in nitric oxide synthase activity in the ovary of gonadotropin treated rats: the role of nitric oxide during ovulation. Endocr J.

[B43] Grazul-Bilska AT, Navanukraw C, Johnson ML, Arnold DA, Reynolds LP, Redmer DA (2006). Expression of endothelial nitric oxide synthase in the ovine ovary throughout the estrous cycle. Reproduction.

[B44] Mitsube K, Zackrisson U, Brannstrom M (2002). Niric oxide regulates ovarian blood flow in the rat during the periovulatory period. Hum Reprod.

